# An Unintended Hazard of Environmental Stewardship: Marine Envenomation Following Invasive Lionfish Culling in Curacao

**DOI:** 10.3390/tropicalmed11070187

**Published:** 2026-07-07

**Authors:** Gregory D. Hawley, Chu Sandy Wang, Andrea K. Boggild

**Affiliations:** 1 Temerty Faculty of Medicine, University of Toronto, Toronto, ON M5S, Canada; 2 Tropical Disease Unit, Toronto General Hospital, Toronto, ON M5G, Canada; 3 Institute of Medical Science, University of Toronto, Toronto, ON M5S, Canada; 4 Scarborough Health Network, Toronto, ON 3G9, Canada

**Keywords:** adventure travel, Caribbean, diving, invasive species, lionfish, marine envenomation, *Scorpaenidae*, skin and soft tissue injury

## Abstract

Marine envenomations are common non-infectious hazards for travelers. Lionfish, venomous fish native to Indo-Pacific waters, have become an invasive species in the Atlantic Ocean and threat to native marine ecosystems. Various control measures have been implemented in response to rapidly expanding lionfish populations, including licensed culling by recreational divers. We herein review lionfish envenomation through framing with a case that occurred during a diving trip to Curacao for the purpose of lionfish spearfishing. Following initial management in Curacao with hot water immersion, wound care, and antibiotic prophylaxis, the patient continued to have persistent swelling, bruising, and pain to the puncture site and was referred to our outpatient clinic for further evaluation. In addition to reviewing clinical syndromes and approach to management for common marine envenomations that may be encountered in the post-travel setting, we situate this case within the broader ecological context of expanding invasive species ranges with climate change and rising sea temperatures. Pre-travel providers should counsel patients at high risk for marine envenomations on preventative measures, along with how and when to seek care following exposure. Post-travel providers should be familiar with the immediate and long-term sequelae of non-infectious envenomations and intoxications, including marine exposures. Larger national and multinational collaborations are required to mitigate the effects of climate change and international marine movement on invasive species, especially those that incur risk to marine and human health alike.

## 1. Introduction

Lionfish, *Pterois* spp. of the family *Scorpaenidae*, are venomous fish species native to Indo-Pacific coastal waters. Lionfish of the *Pterois volitans*/*Pterois miles* complex, have become threatening invasive species in the Atlantic Ocean and represent a major threat to native marine ecosystems [1]. The introduction of lionfish most likely occurred through either intentional or accidental release of aquaria off the coast of Florida in the 1980s [2,3,4]. Since that time, there has been a drastic expansion of lionfish populations throughout the eastern United States seaboard, Caribbean, Gulf of Mexico, and northern coast of South America [5,6,7,8]. Lionfish are now permanently established in these locations, often at much higher densities than in their native habitats, with devastating effects on native fish populations, species richness, and local reef ecosystems [2,5,9,10,11,12,13,14,15,16]. Control of these invasive marine species has become paramount, with several regions in the Caribbean and Americas implementing lionfish culling by licensed divers to help control populations and encourage the regrowth of native coral and marine species [5,9,11,13,14,17,18,19,20,21,22,23,24].

With the emergence of spearfishing as a component of lionfish population control efforts, divers are at increasing risk of lionfish stings. Review of the American Association of Poison Control Center’s (AAPCC) National Poison Data System revealed 8517 reports of aquatic envenomations over a ten-year period in the United States, with 5159 envenomations attributed to fish, although these were not stratified by type of fish [25]. A two-year prospective study in Martinique identified 117 patients with lionfish stings, with 47% of cases occurring in divers [26]. The most common sting location was the upper limb (67%). A public online questionnaire of self-reported lionfish stings identified 555 unique cases, with all included sting events occurring within ten years of the questionnaire completion date [27]. Most individuals reported being in the United States at the time of their lionfish sting (67.03%), with most other cases reported from the Caribbean and Central America. The most common activities implicated in lionfish stings were spearfishing (72.07%) and diving/snorkeling (14.95%), with the majority of individuals reporting a sting to the hand/arm (90.63%). Furthermore, 86.67% of respondents reported being stung underwater, with 85.95% stung by a live fish. Another small-scale questionnaire of fish envenomation identified 67 reports of lionfish envenomation [28]. Scuba diving appeared to be a high-risk activity for lionfish stings; of the 56 incidences of aquatic envenomation related to scuba diving, 54 were attributed to lionfish stings. The most common site of sting was again the hand, although this was not stratified by fish type. There are currently no large scale, standardized incidence reports of lionfish stings, which limits the appreciation of the true burden of lionfish envenomation. The current literature on lionfish sting epidemiology relies on a limited number of surveys, and large governmental surveillance reports, such as that by the US PCC, do not distinguish by type of fish. Many countries, including Canada, do not have standardized reporting systems for lionfish stings. The true incidence of lionfish envenomation, therefore, likely remains largely underreported, including individuals that do not seek medical care, individuals that are treated by healthcare workers but are not reported to surveillance bodies (due to lack of familiarity or absence of reporting bodies), or cases that are reported but not stratified by fish type in publication of surveillance data.

Here, we review lionfish envenomation through framing with a case of a traveler who participates in yearly scuba diving trips for the purpose of lionfish spearfishing, and who was referred for assessment of complications following a lionfish sting in the Caribbean.

## 2. Case Presentation

**Figure 1:**
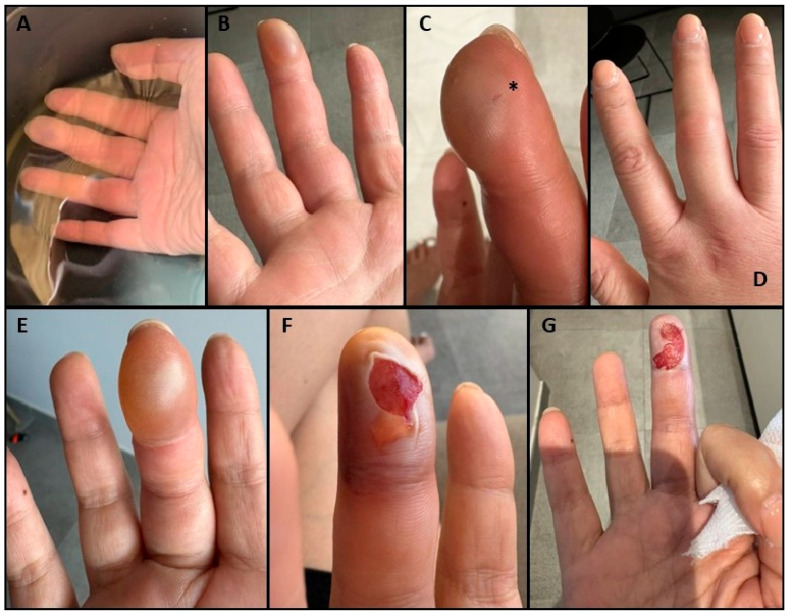
Initial lionfish wound following spine puncture injury. (**A**): hot water immersion following initial sting. (**B**,**C**): Blister formation on day of puncture injury. Puncture wound in Image (**C**) is denoted by an asterisk (*). (**D**): Diffuse edema to the third digit on day of puncture injury. (**E**): Blister formation on day two following puncture injury. (**F**,**G**): Open wound two days after blister incision in Curacao.

A fifty-year old healthy woman, with no significant past medical history or medication use, was referred to our clinic by her Family Doctor for assessment of persistent pain and swelling to the finger following a lionfish sting. She had traveled to Curacao approximately three weeks prior for a scuba diving trip with the purpose of licensed lionfish culling for local population control efforts. While spearfishing, she sustained a puncture injury underwater to the right third finger from the spine of a living lionfish. She promptly removed the spine and proceeded to complete her dive. Following the dive, she was treated with hot water immersion (Figure 1A). The exact temperature and duration of the water immersion were unknown; however, the water felt hot to the touch. Later that day, she developed a large blister to the dorsal pad of her fingertip (Figure 1B,C), along with edema of the entire finger and pain that worsened over the next two days (Figure 1D,E). She did not develop any systemic, respiratory, or cardiovascular symptoms. At that time, she sought medical attention in Curacao, where a local physician opened the blister and cleaned the wound (Figure 1F,G). The patient recalls that clear fluid drained from the blister, without any purulence or visibly retained foreign body. She was prescribed a course of oral azithromycin for wound prophylaxis. Tetanus toxoid was not given, as she was up to date, having received tetanus, diphtheria, and pertussis (TdaP) vaccine several months prior. She completed several further lionfish culling dives along the islands of the Leeward Antilles during the remainder of her trip. She ate the caught lionfish on several occasions following her dives. She returned to Canada approximately two weeks following the envenomation incident, at which time she experienced persistent edema, hyperesthesia, and erythema of her finger. She saw her Family Doctor and was referred to our clinic for assessment.

When seen for initial consultation in our outpatient clinic, she reported ongoing swelling, erythema, bruising, and a small scab at the site where the blister was incised in Curacao. She denied expanding erythema, active drainage, or worsening pain. She denied substantial functional limitations but endorsed a slight deficit in dexterity of the affected finger. Her job required frequent typing, and she noted worsening swelling and pain in the affected finger at the end of the workday. She remained systemically well without any respiratory, cardiovascular, or gastrointestinal symptoms. She denied the occurrence of rash. Aside from the mild hyperesthesia of the fingertip, she denied neurological symptoms, insomnia, or temperature inversion. On assessment, there was a two-millimeter scab on the dorsal surface of the right third fingertip. There were mild erythema, ecchymosis, and edema to the fingertip, with slight tenderness to palpation. There was no open wound, fluctuance, or discharge. Digital pulses and distal sensation to light touch were intact. Her clinical examination was consistent with a lionfish envenomation, with evidence of local reaction secondary to inoculated toxin and possible thermal injury from HWI. There was no evidence of secondary infection or retained foreign body. There was no evidence of complications associated with the cytolytic nature of lionfish venom, such as necrosis or digital ischemia. As such, management at that time was supportive and included ergonomic adjustments, oral analgesics, and protection of the lesion from trauma.

**Figure 2:**
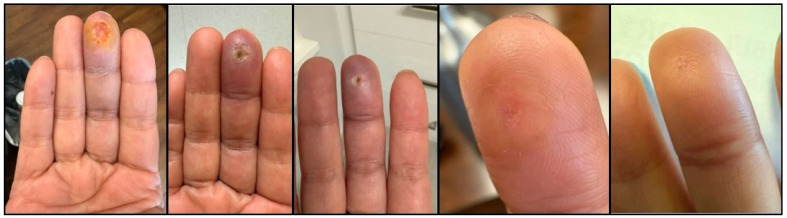
Progressive healing of the lionfish sting, with interval scab formation and healing, and resolution of erythema and ecchymosis. Final image (far right) was taken on two-month re-assessment in our outpatient clinic.

Over the course of two months, she had complete resolution of the edema, erythema, and ecchymosis of the right third fingertip. Her pain resolved, but she continued to experience a residual altered sensation to the fingertip with any form of pressure, such as typing. On examination, there remained a small subcentimeter indentation corresponding to the site of the sting, with complete resolution of erythema and ecchymosis (Figure 2—healing of the puncture wound).

At her final follow-up several months following the original envenomation event, her symptoms had completely resolved with no residual local symptoms or need for ergonomic supports or activity restrictions.

## 3. Discussion

Lionfish stings are an increasingly recognized cause of marine envenomation [29,30]. Lionfish belong to the family *Scorpaenidae*, and are closely related to another well-known venomous fish, stonefishes (family *Synanceiidae*) [31]. In response to perceived threats, lionfish erect dorsal, pelvic, and anal spines capable of penetrating human skin and injecting venom [32]. Across *Scorpaenidae*, venoms are predominantly composed of heat-labile pore-forming cytolysins that are highly conserved within the family [33]. These cytolysins oligomerize within target cell membranes to form non-selective transmembrane pores, resulting in rapid membrane depolarization, and ultimately cell lysis. The primary result of this is local tissue injury with release of intracellular ions which drive an inflammatory response and intense pain [34]. This mechanism is consistent with the evolutionary role of *Scorpaenidae* venoms as a defensive mechanism, where rapid induction of pain serves as the principal deterrent to predation. Additional venom components, including phospholipase and hyaluronidase-like proteins, may lead to further tissue breakdown and amplify local inflammation [34,35,36].

Lionfish possess 12–13 dorsal spines, two pelvic spines, and three anal spines, all of which can release venom [18]. Envenomation typically produces rapid onset, severe, localized pain, often described as sharp, and may radiate peripherally from the puncture site. Pain usually peaks within an hour, but may persist for hours to days, and in the absence of treatment can occasionally last weeks [27,37]. Local swelling, erythema, and inflammation are common. Systemic manifestations are rare but may include sensory changes and, in severe cases, cardiovascular collapse [18,26,38]. Given that species within the *Scorpaenidae* and *Synanceiidae* families share structurally similar cytolysins, the clinical severity of envenomation across species is primarily secondary to venom yield and the efficiency of mechanical delivery systems. While lionfish stings commonly manifest with local pain and edema, stonefish envenomation has the potential for more severe, albeit still rare, complications including tissue necrosis and life-threatening systemic cardiovascular collapse. These differences largely reflect anatomical variation. Stonefish species contain more complex venom delivery apparati and larger venom reservoirs, allowing for the deeper injection of a significantly higher volume of venom per sting [39,40,41].

Management is primarily supportive. The mainstay of therapy is immersion of the affected area in hot water (approximately 45 °C) for 30–90 min [42]. Despite its widespread use as a generally low-risk, first-line intervention for marine envenomation, the evidence supporting HWI remains in debate [43]. One proposed mechanism of action is primarily based on denaturation of heat-labile venom proteins and disruption of the pore-forming toxin structure, potentially reducing their biological activity at the site of envenomation [39,44,45]. Another theory posits that thermal exposure may exert a direct neuromodulatory effect on nociceptors, leading to activation and subsequent desensitization of temperature-sensitive transient receptor potential channels involved in pain signaling pathways [46]. Optimal temperature thresholds, duration of immersion, and timing relative to envenomation are not standardized across the literature, and precise mechanisms of action and evidence-based protocols remain areas of ongoing study [42]. In addition to HWI, meticulous wound care, including wound irrigation, tetanus prophylaxis, and monitoring for secondary bacterial infections should be implemented. Foreign bodies (e.g., spine fragments) should be initially removed mechanically, and imaging studies may be required to further evaluate for retained foreign bodies, which should be removed surgically if symptomatic. If secondary bacterial infection is suspected, antimicrobial regimen selection should include coverage for common marine organisms including *Vibrio vulnificus*, *Aeromonas hydrophila*, and *Shewanella* spp. [47]. In two studies, secondary local infection following lionfish sting was reported in 18/555 (3.24%) [27] and 21/117 (17.95%) [26] cases. Persistent non-response to antimicrobials targeting the aforementioned organisms should raise the possibility of atypical mycobacterial infection and warrants tissue biopsy with culture and susceptibility testing, and possibly molecular diagnostics. Hypotension, if present, should be managed with vasopressors. Late complications include prolonged wound healing, granulomatous inflammation, and persistent sensory changes [48].

*Scorpaenidae* envenomation represents only one type within a broader spectrum of marine envenomation. Other envenomating marine species include cnidarians (e.g., box jellyfish, Portuguese man-of-war, and Irukandji jellyfish), echinoderms (e.g., sea urchins and crown-of-thorns starfish), and mollusks (e.g., cone snails and blue-ringed octopus). Their ecological niches and clinical presentations vary, ranging from localized soft tissue injury to systemic syndromes with potentially fatal neurotoxic and cardiotoxic effects [48,49]. Mainstay of treatment for all marine envenomation is supportive, including HWI. Antivenoms are usually not widely available outside regions to which these species are endemic. Key characteristics, endemic regions, and management strategies for select marine envenomating animals are summarized in Table 1.

Globally, marine envenomation is an underrecognized and underreported cause of human morbidity and mortality. An estimated 40,000–50,000 marine envenomations occur annually worldwide [50]. In Canada, cases are largely imported, and the true incidence of cases remains unknown, as there is no national reporting or surveillance system. Although venomous marine species are most often encountered in tropical regions, climate change, increasing coastal inhabitation, and increased travel and recreational water use all increase the likelihood of human exposure to potential marine envenomation [51,52].

Rising sea temperatures due to climate change promote conditions for venomous species to thrive. For example, jellyfish demonstrate temperature-dependent reproductive growth [53]. The Irukandji jellyfish, once thought restricted to northern Australia, has now been reported in Southeast Asia, Hawaii, the Caribbean, and Florida [49]. Similarly, lionfish, previously endemic only to the Indo-Pacific, have spread across the Western Atlantic, Caribbean, and Mediterranean, where they are now a major invasive species. Warming waters allow for expansion into new marine ecosystems and foster rapid population growth, further expanding the range and density of lionfish that already lack natural predators in these areas [7,11]. Sea urchin and crown-of-thorns starfish populations are also expanding, driven by ocean warming and coral reef degradation [38,54]. These ecological changes all increase the likelihood of human contact with venomous marine organisms.

## 4. Conclusions

Cases of marine envenomation will inevitably be encountered more frequently by health care providers in North America. A thorough history of marine exposures is essential, especially when evaluating injuries in travelers with severe pain or systemic symptoms. Health care facilities should ensure clinician competency in recognition and management of marine envenomation syndromes, particularly given the risk of intoxication syndromes and retention of foreign bodies. For travelers participating in activities that will knowingly place them in direct contact with venomous marine species, such as lionfish culling, pre-travel preparations should include counseling on safety precautions, along with instructions on when and how to seek care following marine injury. Future research in this area should prioritize elucidating the rapidly changing epidemiology of marine envenomations and further exploring potential targeted preventive clothing and apparati, as well as experimental therapies and antivenoms.

## Figures and Tables

**Table 1:** **Overview of common marine envenomations that may be encountered in the post-travel setting by front-line clinicians**.

Organism/Animal	Endemic Region(s)	Mechanism(s)	Clinical Presentation	Management and Other Notes
**Jellyfish** (Cnidaria—e.g., *Chironex fleckeri* (box jellyfish), Portuguese man-of-war, Irukandji)	**Box jellyfish:** Northern coastal waters of Australia, Papua New Guinea, Philippines, Southeast Asia **Irukandji:** Tropical waters of northern Australia **Portuguese man-of-war:** Warmer waters of the Atlantic, Pacific, and Indian Oceans	Nematocysts inject toxins: neurotoxic, cardiotoxic, cytolytic	Rapid onset burning pain, linear “whip-like” erythematous lesions, edema Severe cases: circulatory collapse, respiratory failure	Supportive. Hot water immersion for pain
**Stonefish** **(*Synanceia* spp.)**	Coastal waters of the Indo-Pacific region	Venomous dorsal spines. Proteinaceous venom: cytolytic, neurotoxic, cardiotoxic	Rapid onset pain, local swelling, cyanosis Severe cases: weakness, paralysis, arrhythmias, hypotension	Antivenom not routinely available in Canada but may be imported. Hot water immersion for pain
**Lionfish/Scorpionfish (*Pterois* spp.)**	Indo-Pacific region. Invasive species in Caribbean and Atlantic Ocean.	Venomous spines	Intense pain, edema, erythema, vesicles Severe cases: dizziness, cardiovascular/neuro symptoms	Hot water immersion for pain. Monitor for secondary infection
**Stingray**	Worldwide temperate and tropical coastal waters	Venomous tail spine and puncture trauma	Severe pain, bleeding wound, edema, muscle cramps. Severe cases: hypotension, syncope, arrhythmia	Hot water immersion for pain. Retained spine fragments lead to risk of secondary bacterial infection
**Sea Urchin**	Global ocean range	Sharp spines, venom in some species	Local puncture wounds, pain, swelling, erythema, black/purple discoloration if spine retained Severe cases: weakness, paralysis	Risk of granulomatous foreign-body reaction. Remove spines if possible
**Cone Snail** **(*Conus* spp.)**	Predominantly Indo-Pacific region	Conotoxins (block voltage-gated ion channels)	Local numbness, pain, swelling Severe cases: weakness, paralysis, respiratory failure, coma, death (depending on species)	Supportive care. Punctures can be fatal. No antivenom available
**Blue-ringed Octopus (*Hapalochlaena* spp.)**	Rocky shores and waters between Australia and through the Pacific Ocean north to Japan	Tetrodotoxin (block sodium channels)	Initially painless or mild bite with rapid perioral numbness, weakness, paralysis, respiratory failure	Supportive care including mechanical ventilation. Can be fatal. No antivenom available
**Crown-of-thorns starfish** **(*Acanthaster planci*)**	Indo-Pacific region	Venomous spines with saponins	Painful puncture, swelling, erythema Severe cases: nausea, vomiting, joint pain; delayed wound healing	Supportive care. Risk of secondary bacterial infection

## Data Availability

All data are presented in the manuscript.

## References

[1] del Río L., Navarro-Martínez Z.M., Cobián-Rojas D., Chevalier-Monteagudo P.P., Angulo-Valdes J.A., Rodriguez-Viera L. (2023). Biology and ecology of the lionfish *Pterois volitans/Pterois miles* as invasive alien species: A review. PeerJ.

[2] Hare J.A. (2003). Whitfield PE an Integrated Assessment of the Introduction of Lionfish (*Pterois volitans*/Miles Complex) to the Western Atlantic Ocean. National Oceanic and Atmospheric Administration. NOAA Technical Memorandum NOS NCCOS 2. https://repository.library.noaa.gov/view/noaa/17793/noaa_17793_DS1.pdf.

[3] Ruiz-Carus R., Matheson R.E., Roberts D.E., Whitfield P.E. (2006). The western Pacific red lionfish, *Pterois volitans* (Scorpaenidae), in Florida: Evidence for reproduction and parasitism in the first exotic marine fish established in state waters. Biol. Conserv..

[4] Semmens B.X., Buhle E.R., Salomon A.K., Pattengill-Semmens C.V. (2004). A hotspot of non-native marine fishes: Evidence for the aquarium trade as an invasion pathway. Mar. Ecol. Prog. Ser..

[5] Frazer T.K., Jacoby C.A., Edwards M.A., Barry S.C., Manfrino C.M. (2012). Coping with the lionfish invasion: Can targeted removals yield beneficial effects?. Rev. Fish. Sci..

[6] Johnston M.W., Purkis S.J. (2011). Spatial analysis of the invasion of lionfish in the western Atlantic and Caribbean. Mar. Pollut. Bull..

[7] Schofield P.J. (2009). Geographic extent and chronology of the invasion of non-native lionfish (*Pterois volitans* [Linnaeus 1758] and *P. miles* [Bennett 1828]) in the Western North Atlantic and Caribbean Sea. Aquat. Invasions.

[8] Schofield P.J. (2010). Update on geographic spread of invasive lionfishes (*Pterois volitans* [Linnaeus, 1758] and *P. miles* [Bennett, 1828]) in the Western North Atlantic Ocean, Caribbean Sea and Gulf of Mexico. Aquat. Invasions.

[9] Arias-González J.E., González-Gándara C., Luis Cabrera J., Christensen V. (2011). Predicted impact of the invasive lionfish *Pterois volitans* on the food web of a Caribbean coral reef. Env. Res..

[10] Côté I.M., Maljković A. (2010). Predation rates of Indo-Pacific lionfish on Bahamian coral reefs. Mar. Ecol. Prog. Ser..

[11] Côté I.M., Green S.J., Hixon M.A. (2013). Predatory fish invaders: Insights from Indo-Pacific lionfish in the western Atlantic and Caribbean. Biol. Conserv..

[12] Green S.J., Akins J.L., Maljković A., Côté I.M. (2012). Invasive lionfish drive Atlantic coral reef fish declines. PLoS ONE.

[13] Muñoz R.C., Currin C.A., Whitfield P.E. (2011). Diet of invasive lionfish on hard bottom reefs of the Southeast USA: Insights from stomach contents and stable isotopes. Mar. Ecol. Prog. Ser..

[14] Rocha L.A., Rocha C.R., Baldwin C.C., Weigt L.A., McField M. (2015). Invasive lionfish preying on critically endangered reef fish. Coral Reefs.

[15] Lesser M.P., Slattery M. (2011). Phase shift to algal dominated communities at mesophotic depths associated with lionfish (*Pterois volitans*) invasion on a Bahamian coral reef. Biol. Invasions.

[16] Darling E.S., Green S.J., O’Leary J.K., Côté I.M. (2011). Indo-Pacific lionfish are larger and more abundant on invaded reefs: A comparison of Kenyan and Bahamian lionfish populations. Biol. Invasions.

[17] Albins M.A., Hixon M.A. (2013). Worst case scenario: Potential long-term effects of invasive predatory lionfish (*Pterois volitans*) on Atlantic and Caribbean coral-reef communities. Env. Biol. Fishes.

[18] Morris J.A., Akins J.L. (2009). Feeding ecology of invasive lionfish (*Pterois volitans*) in the Bahamian archipelago. Env. Biol. Fishes.

[19] de León R., Vane K., Bertuol P., Chamberland V.C., Simal F., Imms E., Vermeij M.J. (2013). Effectiveness of lionfish removal efforts in the southern Caribbean. Endanger. Species Res..

[20] Côté I.M., Akins L., Underwood E., Curtis-Quick J., Green S.J. (2014). Setting the record straight on invasive lionfish control: Culling works. PeerJ Prepr..

[21] UNESCO World Heritage Centere Belize Barrier Reef Reserve System. World Heritage List. https://whc.unesco.org/en/list/764.

[22] (2015). Invasive Lionfish Control Ad-Hoc Committee of the Aquatic Nuisance Species Task Force. National Invasive Lionfish Prevention and Management Plan. Gulf States Marine Fisheries Commission. https://westernregionalpanel.org/wp-content/uploads/2022/07/Lionfish_MgntPlan_2015.pdf.

[23] Johnston M.A., Gittings S.R., Morris J.A. (2015). NOAA National Marine Sanctuaries Lionfish Response Plan (2015–2018). NOAA Office of National Marine Sanctuaries. https://sanctuaries.noaa.gov/science/conservation/lionfish15.html.

[24] Ocean Risk and Resilience Action Alliance Invasive lionfish management, Quintana Roo, Mexico—INVERSA Leathers. https://oceanriskalliance.org/project/invasive-lionfish-management-quintana-roo-mexico-inversa-leathers/.

[25] Kirchberg T.N., Cantrell F.L., Coffey C.H., Tomaszewski C. (2024). Nationwide aquatic envenomations reported to US Poison Control Centers from 2011 to 2020. Wilderness Env. Med..

[26] Resiere D., Cerland L., De Haro L., Valentino R., Criquet-Hayot A., Chabartier C., Kaidomar S., Brouste Y., Bruno Mégarbane B., Mehdaoui H. (2016). Envenomation by the invasive *Pterois volitans* species (lionfish) in the French West Indies—A two-year prospective study in Martinique. Clin. Toxicol..

[27] Mouchbahani-Constance S., Choinière M., Sharif-Naeini R. (2023). Understanding the pain experience of lionfish envenomation. Pain. Rep..

[28] Harris R.J., Saggiomo S.L., Paxton G., Motti C.A. (2025). Sting stories: Firsthand experiences of fish envenomation through a small-scale questionnaire. Toxins.

[29] Raechel K., Catherine P., Angel Y. (2024). A systematic review of reports on aquatic envenomation: Are there global hot spots and vulnerable populations?. J. Venom. Anim. Toxins Incl. Trop. Dis..

[30] Cheung W.W.L., Watson R., Pauly D. (2013). Signature of ocean warming in global fisheries catch. Nature.

[31] Rafferty J. Stonefish. Encyclopedia Britannica.

[32] Galloway K.A., Porter M.E. (2021). Predator–prey interactions examined using lionfish spine puncture performance. Integr. Org. Biol..

[33] Díaz C., Angulo A., Brenes O., Ortiz N. (2026). Eastern Pacific scorpionfish *Scorpaena mystes* and *Scorpaena plumieri* (Perciformes: Scorpaenidae) from the coast of Brazil: Two geminate species with very similar venoms. J. Fish. Biol..

[34] Campos F.V., Fiorotti H.B., Coitinho J.B., Figueiredo S.G. (2021). Fish cytolysins in all their complexity. Toxins.

[35] Haddad Junior V., Lopes-Ferreira M. (2023). Envenomations caused by fish in Brazil: An evolutionary, morphological, and clinical vision of a neglected problem. Rev. Soc. Bras. Med. Trop..

[36] Rodriguez C., Carrasco J., Bruner-Montero G., Pires Júnior O.R., Gutiérrez M., Díaz-Ferguson E. (2025). Components and biological activities of venom from lionfishes (Scorpaenidae: *Pterois*). Mar. Drugs.

[37] Diaz J.H. (2015). Marine Scorpaenidae envenomation in travelers: Epidemiology, management, and prevention. J. Travel. Med..

[38] Hobday D., Chadham P., Din A., Geh J. (2016). Denaturing the lionfish. Eplasty.

[39] Church J.E., Hodgson W.C. (2002). The pharmacological activity of fish venoms. Toxicon.

[40] Ziegman R., Alewood P. (2015). Bioactive components in fish venoms. Toxins.

[41] Isbister G.K. (2001). Venomous fish stings in tropical Northern Australia. Am. J. Emerg. Med..

[42] Atkinson P.R.T. (2006). Is hot water immersion an effective treatment for marine envenomation?. Emerg. Med. J..

[43] Carrette T.J., Seymour J.E., Cullen P., Peiera P.L., Little M. (2002). Temperature effects on box jellyfish venom: A possible treatment for envenomed patients?. Med. J. Aust..

[44] Kizer K.W. (1985). Scorpaenidae envenomation. JAMA.

[45] Niżnik Ł., Jabłońska K., Orczyk M., Orzechowska M., Jasińska J., Smoliniec B., Hućko A., Kosowicz P., Klocek A., Słoma P. (2024). Hot-water immersion (HWI) or ice-pack treatment (IPT) as first aid for human envenomation by marine animals? Review of literature. Toxins.

[46] Caterina M.J., Schumacher M.A., Tominaga M., Rosen T.A., Levine J.D., Julius D. (1997). The capsaicin receptor: A heat-activated ion channel in the pain pathway. Nature.

[47] Diaz J.H. (2014). Skin and soft tissue infections following marine injuries and exposures in travelers. J. Travel. Med..

[48] Vetrano S.J., Lebowitz J.B., Marcus S. (2002). Lionfish envenomation. J. Emerg. Med..

[49] Tibballs J., Li R., Tibballs H.A., Gershwin L.A., Winkel K.D. (2012). Australian carybdeid jellyfish causing “Irukandji syndrome”. Toxicon.

[50] Hornbeak K.B., Auerbach P.S. (2017). Marine envenomation. Emerg. Med. Clin..

[51] Purcell J.E., Uye S.I., Lo W.T. (2007). Anthropogenic causes of jellyfish blooms and their direct consequences for humans: A review. Mar. Ecol. Prog. Ser..

[52] Boero F. Review of Jellyfish Blooms in the Mediterranean and Black Sea. Food and Agriculture Organization of the United Nations; 2013. Studies and Reviews, General Fisheries Commission for the Mediterranean No. 92. https://www.fao.org/4/i3169e/i3169e.pdf.

[53] Richardson A.J., Bakun A., Hays G.C., Gibbons M.J. (2009). The jellyfish joyride: Causes, consequences and management responses to a more gelatinous future. Trends Ecol. Evol..

[54] Pratchett M., Uthicke S. (2017). Biology, Ecology and Management of Crown-of-Thorns Starfish.

